# Finite element analysis of stress allocation for designing different root canal

**DOI:** 10.6026/9732063002001175

**Published:** 2024-09-30

**Authors:** Saumyakanta Mohanty, Abhaya Chandra Das, Rashmi Rekha Mallick, Pallabi Choudhury, Priyanka Sarangi, Purobi Choudhury, Nihar Ranjan Sahoo, Sneha Arpana Minz

**Affiliations:** 1 Department of Conservative Dentistry & Endodontics, SCB Dental College & Hospital, Cuttack, Odisha - 753007, India; 2 Department of Periodontics and Oral Implantology, Institute of Dental Sciences, Siksha 'O' Anusandhan (Deemed to be University), Khandagiri Square, Bhubaneswar - 751030, Odisha, India; 3 Lecturer, Government Dental College, Silchar, Srimanta Sankardeva University of Health Science's, Assam, India; 4 Department of Dentistry, Silchar Medical College and Hospital, Attached faculty of Government Dental College, Silchar, Assam; 5 Department of Dentistry, MKCG Medical College Berhampur, Odisha - 760004, India; 6 Intern, Kalinga Institute Dental Sciences, KIIT, Deemed to be University, Bhubaneswar, Odisha, India

**Keywords:** Endodontic therapy, load, root fracture, finite element analysis, stress

## Abstract

It is known that vertical root fractures are influenced by the canal diameter. Therefore, it is of interest to evaluate the stress distribution in three distinct root canal taper designs using finite element analysis. We used a Hyflex Nickel Titanium (NiTi) rotary file to clean and shape a few lower incisor teeth with single canals. Three designs were developed, including a 4% tapered canal preparation, a 6% tapered canal preparation and an 8% tapered canal preparation. Every tooth sample underwent cone beam computed tomography (CBCT) scans and any stresses were found using finite element analysis. The collected data was statistically analysed. In all three designs, the coronal area had the most stress, followed by the middle and the apical area with the least. Enamel had a higher stress value than dentin (MPa). In enamel and dentin with either oblique or vertical stress loading, design 3 (8% taper) had the highest stress value, followed by design 2 (6% taper) and design 1 (4% taper). The difference was statistically significant. Enamel had the highest Young modulus value followed by dentin and mandibular alveolar bone, while periodontal ligament (PDL) had the lowest value. In comparison to the apical and middle part, all canal preparations showed greatest enamel stress at the coronal load locations. As canal tapering rises, so does the stress.

## Background:

The goal of endodontic therapy is for the tooth to be retained after full healing. One major cause of endodontic failure is vertical root fracture (VRF). It has long been established that the prepared canal diameter influences the likelihood of vertical root fractures. Avoidance of root fracture is critical because of the poor diagnosis, prognosis and greater prevalence of Vertical root fracture [1]. Rotary system promotes canal debridement and tapering of instrument, which results into superior cleanliness of root canal wall and lessens the fears about microbial elimination of canal walls [2]. It has been found that root canal tapering reduces the extrusion of root canal irrigants. In reducing the level of Enterococcus faecalis, chemo-mechanical instrumentation found to be effective especially with increase in canal tapering to 8% from 4% [3]. As found in many cases that due to increased tapering of instruments, excessive radicular dentin removal may occur. For the proper pluggers and spreaders penetration, there should be adequate taper [4]. However, excessive taper can also lead to procedural errors. It is also mentioned that chances of root fracture can results when operator excessively remove tissue from canal walls [5]. The location and direction of a root fracture can be influenced by the external root morphology, dentin thickness and the shape of the root canal [6]. Dentin thickness, external root morphology and root canal shape can all affect the direction and location of a root fracture. Taper in general should be adequate to allow deep penetration of pluggers or spreaders during obturation but not so great that procedural errors occur [7]. Several techniques, including finite element analysis, strain gauges, universal testing machines, and microscopes, can be used to assess a tooth's resistance to fracture and stresses [7]. Methods that help in creating finite element analysis (FEA) models are micro computed tomography and cone beam computed tomography (CBCT) [7,8,9]. The elements that affect a fracture's susceptibility were found using finite element analysis [1]. In light of this; we tried the current study to use finite element analysis to ascertain the stress distribution in three tapers of root canal preparation. Null hypotheses were stated prior to study that there was no changes in stress distribution with preparation of variant root canal tapering.

## Materials and Methods:

This in vitro research was conducted in Conservative dentistry department of by single trained investigator. The study was done from 2021 April to 2022 August.

## Inclusion and exclusion criteria:

Inclusion criteria were; mandibular incisors with single canal and matured apex, tooth extracted due to therapeutic reason, absence of any pathology and cracks. Exclusion criteria were teeth with fracture, curved and calcified canals.

## Methods:

Three mandibular incisors with a single canal and comparable exterior morphology were chosen in total. Canals were formed using Hyflex Nickel Titanium (NiTi) rotary files following the provision of access. The 10 K file was used to calculate the working length (WL). Three designs were developed, including a 4% tapered canal preparation, a 6% tapered canal preparation, and an 8% tapered canal preparation. 3 % copious irrigation of sodium hypochloride (NaOCl) was done during the procedure. Following this, we obtained CBCT scan of all teeth samples using Newtom CBCT machine (Newtom GiANO HR,Villa, New Delhi, India) operating at 70 kVp, 12 mAs and 18 seconds exposure. The Cosmos software package (solid works software package, Dassault system Codex, France) was used to import all of the models for meshing. The tetrahedral element was selected from among the various finite elements that made up the finite element model. The elastic parameters (young's modulus and Poisson's ratio) were defined for different tooth sections, access restoration, obturating materials, and neighbouring anatomical components (Table 1).

3D finite element analysis models were created using the identical exterior morphology of the lower incisor overlaid with invariant tapers of the root canal preparation. These models were assigned a force of 100N at 45 degrees to the tooth's long axis and 70N parallel to it. In the current investigation, the resulting models with three distinct designs were subjected to the calculation of vos misses (VM) similar to stresses using the Hyper Mesh v 11.0 ANSYS R 18.1 software. Following compilation and statistical analysis, a significant p value of less than 0.05 was established for the results.

## Results:

Highest Young modulus value was found in (GPa-Giga pascal)) enamel (84.1) followed by dentin (20.2) and Mandibular alveolar bone (14.4) and least was found in PDL (0.0660) (Table 1). It was discovered that, during apical, middle and coronal loading of oblique and vertical stress in Mpa, the stress level in design 1 was lower than in designs 2 and 3. In all three designs, the coronal area had the most stress, followed by the middle and the apical area with the least. Table 2 shows that the difference was statistically significant (p<0.05) (Table 2) Table 3 indicates the stress value in enamel and dentine for design 1, 2, 3 at oblique and vertical direction. The stress was more enamel compared to dentine and it was more in oblique direction compared to vertical direction for all 3 designs for enamel and dentine. Design 3 had the highest stress value, followed by designs 2 and 1. The difference was statistically considerable (P<0.05).

## Discussion:

The preparation of the root canal system is a crucial stage in endodontic treatment. The amount of hard tissue that remains after an endodontic operation impacts on root fractures [10-12]. The likelihood of root fractures increases dramatically in cases where there is less hard tissue [13]. Simultaneously, the augmentation of the coronal third of the root canal space is considered necessary to maintain the measurement of the canal's length, remove debris from the channel, and obdurate the canals [14]. It has been discovered that the root may be weakened by the overuse of rotating tools. It's also clear that going with a thinner taper could reduce the likelihood of root fracture [15-16]. With the aid of finite element analysis; this work ascertains the stress distribution in various tapers of root canal preparation. A universal testing machine is one of the methods of checking stress. It has the advantage of stability, accuracy and control [17]. It is helpful in detecting dentinal defects. The limitation of this method is that it is quite costly and takes longer time. Microscope uses offer finer images of dental structures [18]. Only the taper can exhibit variations in the tooth's anatomy and support to compaction loads, mechanical characteristics, incremental procedures, and temperature profiles, all of which are maintained by the use of finite element analysis (FEA) [19]. According to Reddy *et al.*'s research, the locations of incisal load points were where the peak von Misses stresses (VM) stress occurred on all models. In all three designs, the highest VM stresses were seen in the peri-cervical dentin area [7]. The conclusions align with our findings. Under a 200-N multipoint load, Michael *et al.* found that the Pro-Taper Gold prepared models had the highest VM stress in comparison to the V-Taper 2H prepared models. Peri-cervical dentin showed greatest stress, while the root showed a drop in stress apically [20]. Cheng *et al.* used finite element analysis to compare stress delivery within roots with curved canals prepared using three different methods. The researchers concluded that vertical compaction caused high stress in the region immediately beneath the loading site. In replicated curved canals, proper canal preparation techniques have a minor impact on stress delivery [21]. Complex structures are analysed using the finite element analysis (FEA) numerical engineering method, which takes into account the material properties of the structures. FEA is used to evaluate the distribution and magnitude of stresses under masticatory load when a structure is subjected to force [1]. Using a FEA has the primary advantage of allowing all conditions to be precisely matched. PDL and alveolar bone were added to the FEA models in order to replicate the clinical state [7]. During filling, the root stress reduces as the canal taper increases, with the highest strains created at the apex and along the canal wall. The stress generated is highest at the cervical part of the root surface and increases slightly with increasing taper [1]. The stress load in the current investigation was largest in design 3 (followed by designs 2 and 1), enamel compared to dentine and coronal compared to apical. In all three designs, the oblique load resulted in more stress than the vertical load. The design with 8% canal preparation has more stress than the version with 4% tapers. Therefore null hypotheses were nullified that there was no changes in stress distribution with different root canal tapering preparation. According to the current study, teeth with more taper have weaker structures. Canal preparation should be as conservative as possible while still providing adequate cleaning and shaping to avoid stress distribution and VRF. A canal shape that is smoothly rounded is advantageous and can reduce stress concentration.

## Limitation:

Even though FEA is a versatile and useful method, it has few drawbacks. The precise reproduction of tooth configuration remains a challenge for FEA. Furthermore, the delivery of forces on the canal surface is assumed to be consistent in most FEA experiments. The other limitation of the current study is smaller sample size. Further studies are needed to validate the results.

## Conclusion:

The incisal load locations were where all canal preparations showed the highest levels of enamel stress. As canal taper grew, so did the VM strains. Compared to dentine, enamel had a higher stress load. As canal tapering grows, stress rises. In contrast to a vertical load, the stress was greatest under an oblique force.

## Figures and Tables

**Table 1:** Assessment of Young's modulus and poisson's ratio [10]

**Material**	**Young's modulus GPa**	**Poisson's ratio**
Enamel	84.1	0.331
Dentin	20.2	0.31
Periodontal ligament	0.066	0.42
Mandibular alveolar bone	14.4	0.27

**Table 2:** Peak stress at different tooth locations (Mpa)

**Design**	**Apical**		**Middle**		**Coronal**	
	Oblique	Vertical	Oblique	Vertical	Oblique	Vertical
1	12.1	3.4	26.1	5.14	44.1	7.3
2	21.4	3.6	39.1	7.5	47.5	11.6
3	23.3	4.4	26.5	8.1	39.3	11.2
P value	0.05	0.1	0.01	0.03	0.18	0.05

**Table 3:** Evaluation of the maximum stress values in dentin and enamel (Mpa)

**Design**	**Enamel**		**Dentin**	
	Oblique	Vertical	Oblique	Vertical
1	90.7	46.1	38.7	136
2	109.8	78.8	46.8	16.7
3	111.2	96.7	48.6	28.3
P value	0.05	0.14	0.01	0.05
